# Self-isolation negatively impacts self-management of diabetes during the coronavirus (COVID-19) pandemic

**DOI:** 10.1186/s13098-021-00734-4

**Published:** 2021-10-29

**Authors:** Shahina Pardhan, Md. Saiful Islam, Guillermo F. López-Sánchez, Tirthalal Upadhyaya, Raju P. Sapkota

**Affiliations:** 1 Vision and Eye Research Institute, School of Medicine, Anglia Ruskin University, East Road, Cambridge, CB1 1PT UK; 2 Department of Public Health and Informatics, Jahangirnagar University, Savar, Dhaka-1342 Bangladesh; 3 Department of Medicine, Gandaki Medical College and Teaching Hospital, Pokhara, Nepal

**Keywords:** Diabetes, Self-management, Blood glucose, COVID-19, Mandatory self-isolation

## Abstract

**Background/Aim:**

People with diabetes are at a greater risk of serious complications from Coronavirus disease (COVID-19). Self-management of diabetes is therefore of paramount importance. The purpose of this study is to compare self-management of diabetes pre-COVID-19 and during the COVID-19 pandemic.

**Methods:**

679 participants with diabetes completed an online structured questionnaire survey. Various exposure variables (demographics, duration, treatment and complications of diabetes, self-isolation, etc.) were analysed to examine associations with the following outcome variables: (i) fluctuation of blood glucose levels, (ii) access to diabetes medicine, (iii) access to healthy diet, (iv) physical activity. Adjusted multiple regression analysis ascertained significant associations for each outcome variable against exposure variables.

**Results:**

Multiple regression analysis showed that self-isolation was significantly associated with greater fluctuation in blood glucose levels (*OR* = 1.8, 95% *CI* = 1.2–2.6, *p* = 0.005), reduced access to diabetes medicine (*OR* = 1.9, 95% *CI* = 1.1–3.1, *p* = 0.02) and reduced access to healthy diet (*OR* = 3.0, 95% *CI* = 2.0–4.6, *p* < 0.001). Fluctuation in blood glucose level was also significantly associated with having at least one complication of diabetes (*OR* = 2.2, 95% *CI* = 1.2–3.9, *p* = 0.008) and reduced access to diabetes medicine was significantly higher in people who were on insulin (*OR* = 2.1, 95% *CI* = 1.3–3.3, *p* = 0.001).

**Conclusions:**

Self-isolation was shown to impact almost all factors that influence self-management of diabetes. A targeted approach to improved access to diabetes medicine, healthy diet for people who needed to self-isolate is vital in order to ensure that they are able to self-manage their diabetes effectively.

## Introduction

Since the coronavirus disease was first identified in Wuhan, Hubei province of China in 2019 (named as COVID-19 by the World Health Organisation [WHO], on 11 February 2020), its spread around the world at an alarming rate forced the WHO to declare it as a global health emergency in just within two and half months (on March 11, 2020) from its first detection. So far (as of September 29, 2021), more than 232 million people have been infected by COVID-19 worldwide with over 4.7 million mortalities [1].

The pandemic has affected the lives and livelihood of millions of people forcing them to suddenly adapt to a ‘new normal’ way of living. Amongst the changes brought about by the COVID-19 pandemic include the requirement to wear facemasks, frequent washing of hands, the need to maintain safe physical distance from each other, having to minimise non-essential travel/contact, etc. Furthermore, people have been forced to live under severe restrictions in movement and ban on the social gathering, closure of shops, sporting venues, leisure parks and praying venues, shortage of food supplies and panic buying. The pandemic also has a negative impact on the mental wellness of people as a result of worry around fear of job losses, the possibility of being infected with coronavirus, etc. Changes due to pandemic also included having to work remotely and an increased sedentary lifestyle. In addition, self-isolation is a requirement if infected with COVID-19 or someone living with has tested positive for COVID-19. All these changes can impact upon people’s access to medicine, physical activity, healthy diet and health services including cancellation or postponement of non-urgent appointments [2], leading to poor control of non-communicable disease including diabetes [3–9].

People with underlying diseases such as diabetes are at an increased risk of severe illness and mortality from the COVID-19 infection. A recent meta-analysis of 33 studies found that people with diabetes were 2.7 times more likely to develop severe illness following COVID-19 infection and 1.9 times more likely to die from it compared to those without diabetes [10]. It is therefore important that people with diabetes are able to monitor blood glucose levels regularly, attend doctor/hospital appointments, access the right kind of medicine, be physically active and eat healthy food [11]. Research also suggests that active and proper self-management reduces the risk of serious complications of diabetes by 53–63% and the risk of mortality by 46% [12, 13] as people self-manage their diabetes over 95% of the time [14].

Recent research on people with diabetes has shown increased consumption of carbohydrate and sugary foods, decreased physical activity, reduced self-monitoring of blood glucose levels and increased stress and anxiety during the pandemic [15–19]. On comparing pre-pandemic and during pandemic states, studies have shown greater fluctuation in blood glucose levels in patients with diabetes [20], worsening of complications of diabetes [21] and higher blood glucose levels in newly diagnosed patients [22]; factors associated with these changes are not well understood. On the other hand, studies from India and Poland have reported a positive effect on people’s behaviour and blood glucose levels during the pandemic including more consumption of fruits, vegetables and nutritious/regular meals [15, 23]. A study from Italy reported that blood glucose levels improved during the first seven days of stopping to work suggesting that slowing down of daily routine might have a beneficial effect (at least in the short-term) on diabetes control [24].

A number of studies have examined the effect of COVID lockdown on diabetes self-management [25–28]. These studies found that blood glucose levels fluctuated more during the COVID lockdown and were associated with poor diet, increased anxiety and reduced physical activity levels. Various interventions were suggested such as increased physical activity at home [27, 28] and eating more vegetables [27] to improve blood glucose levels during the lockdown. However, how different factors including (but not limited to) the mandatory need to self-isolate (with strict domiciliary confinements) because someone (or a person living with) was infected with COVID-19, duration of self-isolation, age, gender, duration, treatment and complications of diabetes are associated with diabetes self-management during the COVID pandemic is not well known. Needing to self-isolate means people are strictly not able to go out of their homes, as a consequence of which their basic outdoor activities such as walking, running, cycling, going to the doctor/pharmacy to get medicine and buying healthy foods are likely to get worsened compared to those who did not need to self-isolate as they could still go out of their homes for these basic outdoor activities. These (above) factors, together with anxiety about underlying health (e.g., diabetes) and loneliness because of not being able to socialise face-to-face with friends and relatives may put people with diabetes who needed to self-isolate mandatorily (due to COVID-19 infection or coming in close contact with someone who was infected with COVID-19) at a higher risk of poor diabetes self-management and uncontrolled diabetes, which was investigated in this study. Such as investigation is needed in order that evidence-led guideline development for improved diabetes self-management by targeting those risk factors significantly associated with poor diabetes self-management during (or specific to) the COVID-19 pandemic is possible.

## Methodology

### Study aim

To examine how various exposure factors including demographic characteristics (age, gender), duration, treatment and complications of diabetes, the need to self-isolate (due to being infected or coming in contact with someone infected with COVID-19) and duration of self-isolation (see Table 1), were associated with the following outcomes of diabetes self-management: (i) fluctuation of blood glucose levels; (ii) reduced access to diabetes medicine; (iii) reduced access to right kind of food for diabetes; and (iv) reduced physical activity.

### Study design and setting

A cross-sectional online survey carried out between May 2020 and November 2020.

### Survey material

A structured questionnaire built on an existing validated diabetes self-management questionnaire [29], and previous literature on factors that may affect the management of diabetes including demographics, history of diabetes (duration/treatment and any long-term complications such as retinopathy, neuropathy, nephropathy), access to diabetes medicine, fluctuation in blood glucose levels, access to a healthy diet, and physical activity [8, 9]. Questions around COVID-19 such as whether participants had experienced symptoms or tested positive for COVID-19 by a standard Polymerase Chain Reaction (PCR) test, whether they needed to self-isolate, etc., were adapted from the previous studies [30, 31]. All questions were reviewed and agreed upon by authors (SP and RS) who are experienced in conducting survey-based studies [8, 9, 30], which were then pretested/validated on a small sample of people with diabetes (n = 20) who were not included in this study.

### Power analysis

With a 12% prevalence rate of diabetes in the adult population [31], precision error of 5% and type 1 (α) error of 5%, the required sample size was calculated to be 676. Our sample size exceeds this number.

### Study participants

A total of 679 participants (UK, Bangladesh, India and Nepal, 52% male) above 18 years of age (median age range: 50–60 years) who self-reported to have diabetes (of any type) completed the survey. Work from our laboratory has shown that not all our participants are aware of the type of diabetes they have [9] so we asked them to comment on whether they were on insulin treatment or not which we consider to be more appropriate for the aims of this study (30.4% of the total participants reported being on insulin or combined treatment, Table 1). Also, we did not ask for information about gestational diabetes or maturity onset diabetes of the young (MODY), which is caused by a gene mutation.

Participants were treated in accordance with applicable ethical guidelines that followed the tenets of the Helsinki Declaration. Information that could identify individual participant during or after data collection were not recorded. The study protocol was approved by Anglia Ruskin University’s Faculty of Health, Education, Medicine and Social Care Research Ethics Panel [ref. no.: 19/20/024].

### Procedure

Participants were invited to take part in the study through social media (Facebook, WhatsApp), online adverts, word of mouth, or contacts through acquaintances. Participants were informed at the beginning (i.e., before the survey began) about the purpose of the study and were provided with information about their anonymity and confidentiality. Participants were allowed to complete the survey only once and exit it at any time, but if exited without clicking the ‘submit’ button their responses were not saved. They were required to complete the entire survey in a single session which lasted for approximately 8–10 min. All the questions needed a response before the ‘submit’ button could be enabled. Where participants were not able to complete the survey themselves (for example, due to being computer illiterate), responses were either collected via telephone interviews by a member of the research team in local language or participants could ask their literate family members to complete the survey on their behalf. No face-to-face data collection was performed to avoid the risk of COVID-19 infection or transmission.

All survey responses were anonymous. Participant’s responses across questions could vary from ‘yes’, ‘no’, ‘not sure’, ‘not applicable’, ‘prefer not to say’ etc. The responses were relabelled afterwards into binary forms, e.g., ‘yes’, ‘no/not sure’. Responses like ‘not applicable’ (for example to a specific question, ‘did you have to self-isolate because you tested positive for COVID-19 or had COVID-19 like symptoms?’) were not included in the analyses. Guidelines for conducting survey studies were followed [32].

Exposure and outcome variables were categorised as per the aims of our study and analyses were carried out appropriately. Associations between exposure variables (age, gender, duration of diabetes and type of treatment, known complications of diabetes, and the need for mandatory self-isolation) and each outcome variable (fluctuation of blood glucose levels, change in access to diabetes medicine and healthy diet, and change in physical activity) was examined.

### Data analysis

Bivariate analysis (Chi-square, Fisher’s exact test) examined associations between each exposure and the outcome variable. Significant variables were then fitted into a multivariate logistic regression. Test of multicollinearity was performed for categorical variables, and where the exposure variables were highly correlated (e.g., need to self-isolate, testing positive for COVID-19) only one variable was used in the multivariate logistic regression analysis.

## Results

Out of all the 679 participants, as Table 1 shows, 52% were males, over 61% were ≥ 50 years of age, 57% reported having diabetes for more than 5 years and 8.6% reported they had developed at least one form of long-term complications of diabetes such as the retinopathy. Of the total, 30.4% were on insulin treatment, 20.6% (n = 140) needed to self-isolate because of the COVID-19 infection in themselves or someone living with or experiencing COVID-19 like symptoms such as fever, persistent cough, difficulty breathing and loss of taste and smell. COVID-19 symptoms lasted for more than one week in 62.1% of those who needed to self-isolate.

**Table 1:** Demographics with participant numbers and percentages for different exposure variables

Variable	Categories	Number (%)
Age group	< 50 years	265 (39%)
	50 years or above	414 (61%)
Gender	Male	354 (52.1%)
	Female	325 (47.9%)
Duration of diabetes	Up to 5 years	294 (43.3%)
	> 5 years	385 (56.7%)
Having diabetes complications like retinopathy	Yes	58 (8.6%)
	No	617 (91.4%)
Diabetic Treatment	Insulin/combined	204 (30.4%)
	Non-insulin	466 (69.6%)
Needing to self-isolate mandatorily	Yes	140 (20.6%)
	No/prefer not say	539 (79.4%)
Duration of self-isolation	Up to one week	53 (37.9)
	Over one week	82 (62.1%)
Tested positive for COVID-19 by a standard test	Yes	55 (17.1%)
	No	266 (82.9%)

Rest of the ‘Results’ focus on identifying factors that were associated with different outcomes important for diabetes self-management (A-D, below) during (specific to) the COVID-19 pandemic compared to pre-pandemic.

### Greater fluctuation of blood glucose levels

A total of 242 (35.6%) participants reported that their blood glucose levels fluctuated more during the COVID pandemic compared to the pre-pandemic. Table 2A shows Chi-square results for various exposure variables associated with the greater fluctuation of blood glucose levels during the pandemic.

**Table 2:** Fluctuation of blood glucose levels

A				
		Outcome variable (Greater fluctuation of blood sugar levels) response category		P-value
Exposure variable	Exposure variable response category	Yes	No/not sure	
Age group	< 50 years	105 (38.9%)	165 (61.1%)	0.93
	50 years or above	137 (38.3%)	221 (61.7%)	
Gender	Male	126 (38.9%)	198 (61.1%)	0.87
	Female	116 (38.2%)	188 (61.8%)	
Duration of diabetes	Up to 5 years	105 (38.9%)	165 (61.1%)	0.47
	> 5 years	137 (38.3%)	221 (61.7%)	
Treatment of diabetes	Non-insulin	161 (36.6%)	279 (63.4%)	0.07
	Insulin/combined	80 (43.5%)	105 (56.5%)	
Presence of diabetes complications like retinopathy	Yes	30 (56.6%)	23 (43.4%)	0.005*
	No/not sure	189 (36.9%)	323 (63.1%)	
Needing self-isolation	Yes	65 (50.0%)	65 (50.0%)	0.003*
	No/prefer not say	177 (35.5%)	321 (64.5%)	
Duration of self-isolation	Up to one week	26 (48.1%)	25 (51.9%)	0.44
	More than one week	38 (48.1%)	41 (51.9%)	

B				
Exposure variable	Exposure variable category	Association with greater fluctuation of blood sugar levels		P-value
		Odds Ratio (95% CI)		
Presence of diabetes complications like retinopathy	Yes	2.18	(1.23–3.87)	0.008*
	No/not sure (Reference)			
Needing to self-isolate	Yes	1.76	(1.18–2.61)	0.005*
	No/prefer not say (Reference)			

**A** Chi-square tests showing association of various exposure variables with the outcome variable of greater fluctuation of blood glucose levels during the pandemic compared to pre-pandemic. *represent significant p-values. **B** Adjusted Multivariate Logistic Regression results showing needing to self-isolate and presence of diabetes complications as significant factors associated with greater fluctuation of blood glucose levels during the pandemic compared to pre-pandemic

Multivariate logistic regression showed a greater fluctuation in blood glucose levels was significantly associated with the need to self-isolate (*OR* = 1.8, 95% *CI* = 1.2–2.6, *p* = 0.005) (Table 2B) and also with the presence of at least one form of long-term complication of diabetes such as the retinopathy, neuropathy and nephropathy (*OR* = 2.2, 95% *CI* = 1.2–3.9, *p* = 0.008).

### Reduced access to diabetes medicine

A total of 94 (13.8%) participants reported their access to diabetes medicine got worse during the pandemic compared to the pre-pandemic. Table 3A shows Chi-square results for various exposure variables associated with reduced access to diabetes medicine during the pandemic (compared to pre-pandemic).

**Table 3:** Reduced access to diabetes medicine

A				
		Outcome variable (Reduced access to diabetes medicine) response category		P-value
Exposure variable	Exposure variable category	Yes	No/not sure	
Age group	< 50 years	35 (17.2%)	168 (82.8%)	0.99
	50 years or above	59 (17.4%)	281 (82.6%)	
Gender	Male	48 (16.8%)	237 (83.2%)	0.82
	Female	46 (17.8%)	212 (82.2%)	
Duration of diabetes	Up to 5 years	36 (17.0%)	176 (83.0%)	0.91
	> 5 years	58 (17.5%)	273 (82.5%)	
Treatment of diabetes	Non-insulin	48 (13.4%)	310 (86.6%)	0.001*
	Insulin/combined	46 (25.0%)	138 (75.0%)	
Presence of diabetes complications like retinopathy	Yes	14 (26.9%)	38 (73.1%)	0.08
	No/not sure	79 (16.2%)	409 (83.8%)	
Needing to self-isolate	Yes	28 (25.5%)	82 (74.5%)	0.02*
	No/prefer not say	66 (15.2%)	367 (84.8%)	
Duration of self-isolation	Up to 1 week	8 (21.1%)	30 (78.9%)	0.64
	More than 1 week	19 (26.0%)	54 (74.0%)	

B				
Exposure variable	Exposure variable category	Association with reduced access to diabetes medicine		P-value
		Odds Ratio (95% CI)		
Treatment of diabetes	Insulin or combined	2.13	(1.35–3.35)	0.001*
	Non-insulin (Reference)			
Needing to self-isolate	Yes	1.86	(1.11–3.09)	0.02*
	No/prefer not say (Reference)			

**A** Chi-square results showing association of various exposure variables with the outcome variable of reduced access to diabetes medicine during the pandemic compared to pre-pandemic. *represent significant p-values. **B** Adjusted Multivariate Logistic Regression results showing needing to self-isolate and being on insulin treatment are associated significantly with reduced access to diabetes medicine during the pandemic compared to pre-pandemic

Multivariate logistic regression showed reduced access to diabetes medicine was significantly associated with the need to self-isolate (*OR* = 1.9, 95% *CI* = 1.1–3.1, *p* = 0. 02) (Table 3B) and the use of insulin treatment (*OR* = 2.1, 95% *CI* = 1.3–3.3, *p* = 0.001).

### Reduced access to right kind of food

A total of 142 (20.9%) participants reported that their access to the right kind of food suggested for good diabetes control reduced during the pandemic compared to pre-pandemic. Table 4A shows Chi-square results for various exposure variables associated with the reduced access to healthy food recommended for good diabetes control during the pandemic (compared to pre-pandemic).

**Table 4:** Reduced access to healthy diet recommended for good diabetes control

A				
		Outcome variable (Reduced access to healthy diet) response category		P-value
Exposure variable	Exposure variable category	Yes	No/not sure	
Age group	< 50 years	54 (21.8%)	194 (78.2%)	0.77
	50 years or above	88 (22.9%)	297 (77.1%)	
Gender	Male	68 (20.7%)	261 (79.3%)	0.29
	Female	74 (24.3%)	230 (75.7%)	
Duration of diabetes	Up to 5 years	70 (26.0%)	199 (74.0%)	0.06*
	> 5 years	72 (19.8%)	292 (80.2%)	
Treatment of diabetes	Non-insulin	96 (21.7%)	346 (78.3%)	0.53
	Insulin/combined	45 (24.2%)	141 (75.8%)	
Presence of diabetes complications like retinopathy	Yes	13 (24.5%)	40 (75.5%)	0.73
	No/not sure	128 (22.2%)	448 (77.8%)	
Needing to self-isolate	Yes	50 (52.0%)	75 (48.0%)	< 0.001*
	No/prefer not say	92 (23.4%)	146 (76.6%)	
Duration of self-isolation	Up to one week	25 (53.2%)	22 (46.8%)	0.57
	More than one week	33 (42.9%)	44 (57.1%)	

B				
Exposure variable	Exposure variable category	Association with reduced access to healthy diet		P-value
		Odds Ratio (95% CI)		
Duration of diabetes	Up to 5 years	1.01	(0.68–1.50)	0.6
	> 5 years (Reference)			
Needing to self-isolate	Yes	3.01	(1.97–4.60)	< 0.001*
	No/prefer not say (Reference)			

**A** Chi-square results showing association of various exposure variables with the outcome variable of reduced access to healthy food for good diabetic control during the pandemic compared to pre-pandemic. *represent significant (or near significant) p-values. **B** Adjusted Multivariate Logistic Regression results showing needing to self-isolate is associated significantly with reduced access to healthy food during the pandemic compared to pre-pandemic

Multivariate logistic regression showed that reduced access to healthy food recommended for good diabetes control was significantly associated with the need to self-isolate (*OR* = 3.0, 95% *CI* = 2.0–4.6, *p* < 0.001) (Table 4B).

### Reduced physical activity

A total of 308 (45.4%) participants reported that their overall physical activity reduced during the pandemic compared to pre-pandemic. Table 5 shows Chi-square results for various exposure variables associated with reduced physical activity during the pandemic (compared to pre-pandemic). None of the exposure variables was found to be significantly associated with the outcome variable of reduced physical activity.

**Table 5:** Reduced physical activity

		Outcome variable (reduced physical activitiy) response category		
Exposure variable	Exposure variable category	Yes	No/not sure	P-value
Age group	< 50 years	118 (47.6%)	130 (52.4%)	0.41
	50 years or above	190 (51.1%)	182 (49.9%)	
Gender	Male	150 (46.9%)	170 (53.1%)	0.17
	Female	158 (52.7%)	142 (47.3%)	
Duration of diabetes	Up to 5 years	135 (49.8%)	136 (50.2%)	0.99
	> 5 years	173 (49.6%)	176 (50.4%)	
Treatment of diabetes	Non-insulin	217 (49.8%)	219 (50.2%)	0.99
	Insulin/combined	90 (50.0%)	90 (50.0%)	
Presence of diabetes complications like retinopathy	Yes	26 (55.3%)	21 (44.7%)	0.45
	No/not sure	280 (49.2%)	289 (50.8%)	
Needing to self-isolate	Yes	66 (51.2%)	63 (48.8%)	0.77
	No/prefer not say	242 (49.3%)	249 (50.7%)	
Duration of self-isolation	Up to one week	20 (41.7%)	28 (58.3%)	0.14
	More than one week	45 (56.2%)	35 (43.8%)	

Chi-square results showing association of various exposure variables with the outcome variable of reduced physical activity during the pandemic compared to pre-pandemic. Multivariate Regression analysis was not performed since significant association was found for none of the exposure variable

The outcome variables provided in Table 2 (fluctuation in blood sugar levels) and Table 3 (access to diabetic medicine) are not strictly the self-care behaviors. Nonetheless, the inclusion of these variables respectively provided reflection of whether diabetes was adequately monitored and controlled, and the patients’ compliance with diabetes medication, which are important self-care behaviors [33].

## Discussion and conclusion

Diabetes is one of the most commonly reported comorbidities in people infected with COVID-19 [34–36]. At a time when access to health care facilities is severely limited owing to lockdown/quarantine measures and cancellation or postponement of routine/non-urgent appointments, it has become more important than ever before to examine how various factors influence diabetes self-management during the COVID-19 pandemic. Limited access to medicine and healthcare professionals, fresh food, physical activity, and increased stress and anxiety have been identified as common barriers to good diabetic control during the COVID pandemic [15, 25–28, 37]. In this study, we extend and show how other factors including the need to self-isolate, duration of self-isolation, age, gender, duration, treatment and complications of diabetes are associated with diabetes self-management during the COVID pandemic so that specific barriers to improved diabetes self-management can be targeted.

The prevalence of COVID-19 infection (confirmed by a standard PCR test) was found to be 8.1% in all our participants agreeing with previous studies which have reported rates of 8–10% in people with diabetes in the community [38, 39]. However, among patients hospitalized with COVID-19, a much higher prevalence (19%) of diabetes has been reported [40].

In our total sample, 35.6% reported that their blood glucose levels fluctuated more during the pandemic. In addition, 45.4% of participants reported their physical activity reduced during the pandemic. A study from India in young adults with type 1 diabetes reported deterioration of blood glucose levels in 72% of people and reduced physical activity in 90% of people during the pandemic [41]. Our study agrees with that of Khare and Jindal [26], who reported worsening of blood glucose level in 39.2% of diabetes patients although it was not clear what proportion of these patients were self-isolating.

Needing to self-isolate was found to be the single most important factor influencing all (but reduced physical activity) variables investigated in our study that have the potential to influence diabetes self-management. The likelihood of fluctuation in blood glucose levels was nearly two times higher (*OR* = 1.8*,* 95%, *p* = 0.005, *CI* = 1.2–2.6) in those who needed to self-isolate compared to those who did not need to self-isolate, and over two times higher (*OR* = 2.2*,* 95%, *p* = 0.008, *CI* = 1.2–3.9) in those who had diabetes complications compared to those who did not.

Self-isolation was significantly associated with reduced access to diabetes medicine during the pandemic with odds ratio of nearly two times higher (*OR* = 1.9*,* 95%, *p* = 0.02, *CI* = 1.1–3.1) in those who needed to self-isolate compared to those who did not. In addition, those who were on insulin treatment were over two times more likely to have reduced access to diabetes medicine (OR = 2.1, 95%, *p* = 0.001, *CI* = 1.3–3.3) compared to those who were not on insulin treatment.

Our study adds to the literature in that we show that self-isolation was also significantly associated with reduced access to healthy food and diabetes medicine. A qualitative study from Wuhan, China in patients with Type 2 diabetes (n = 12) who had to self-isolate after being discharged from the hospital for COVID infection identified barriers such as shortage of physical spaces and resources (food, medicine) to be the key factors hindering diabetes self-management [25].

It was somewhat surprising that none of the exposure variables, and in particular those who needed self-isolation, were found to be significantly associated with reduced physical activity during the COVID-19 pandemic. This may be because living either under total or partial lockdown at the time of the survey could have limited the overall outdoor physical activities for most participants and not just for those who needed to self-isolate [18, 27].

Self-management of diabetes involves many more activities (foot care, smoking, adherence to medications, stress management, keeping review dates, etc.) in addition to the parameters we have examined. Inclusion of those parameters would have made the study more comprehensive and can be included in a future study. Also, our study compared self-reported data before and during the pandemic. This retrospective judgement could have been influenced by differences in bias and memory recall abilities. However, the only way around that would have been to collect the data before the pandemic began which was not possible.

While some earlier studies have reported the positive effect of COVID pandemic on behavior of diabetes people such as increased consumption of fruits and vegetables [23] and increased motivation for home exercise [42], several other studies have shown that access to healthy diet and physical exercises in diabetes people deteriorated significantly during the COVID-19 pandemic [15–18]. Our data show that people with diabetes who are required to self-isolate are at a higher odds of poor diabetes self-management (as a result of reduced access to healthy food and diabetes medicine) and uncontrolled diabetes, and need more informed self-management guidelines in order to enable better self-management. In addition, our data also show the need for having improved access to diabetes medicine in those who are on insulin treatment, and increased awareness about self-monitoring of glucose levels in people who have complications of diabetes in order to prevent further serious complications. Public health policies should give priority to those people with diabetes who need to isolate because of COVID-19 infection and also those who have diabetes complications in order to ensure that they are able to manage their diabetes appropriately during the pandemic.

## Data Availability

The datasets generated during and/or analysed during the current study are available from the corresponding author on reasonable request**.**
